# Tracking persistent and resistant *Enterococcus faecalis* and *E. faecium* from farm to fork: biofilm-linked risks in antibiotic resistance of isolates

**DOI:** 10.1007/s11259-025-11061-8

**Published:** 2026-01-15

**Authors:** Kursat Koskeroglu, Nurhan Ertas Onmaz, Dursun Alp Gundog, Candan Gungor, Guven Gungor, Kálmán Imre, Adriana Morar

**Affiliations:** 1 Department of Veterinary Public Health, Faculty of Veterinary Medicine, Erciyes University, 38039 Kayseri, Türkiye; 2 Department of Food Hygiene and Technology, Faculty of Veterinary Medicine, Yozgat Bozok University, Yozgat, Türkiye; 3 Department of Biostatistics, Faculty of Veterinary Medicine, Bingöl University, Bingöl, 12000 Türkiye; 4 Department of Animal Production and Veterinary Public Health, Faculty of Veterinary Medicine, University of Life Sciences ‘‘King Mihai I’’ from Timisoara, Timișoara, Romania

**Keywords:** E. faecium, E. faecalis, Farm-to-fork continuum, Antibiotic resistance, Biofilm formation, Food safety

## Abstract

This study aimed to investigate the prevalence, antimicrobial resistance, biofilm-forming ability, virulence gene profiles, and associated risk levels of *Enterococcus faecalis* and *E. faecium* isolated along the farm-to-fork meat production continuum in Kayseri, Türkiye. Out of 348 samples analyzed, *Enterococcus* spp. were detected in 209 (60%) of the samples, of which 41 (20%) were *E. faecalis* and 48 (23%) were *E. faecium.* Both strains were resistant to at least one antimicrobial agent, and 35 isolates (39%) exhibited multidrug resistance (MDR). Among the tested antibiotics, resistance rates were particularly high for tetracycline (66% in *E. faecalis*, 69% in *E. faecium*) and erythromycin (56% and 58%, respectively); resistance to vancomycin (10% in each species) and ciprofloxacin (12% in *E. faecalis* and 13% in *E. faecium*) was low but consistently occurred in combination with resistance to other antibiotics and exclusively within multidrug resistance patterns. All isolates formed biofilms, with 55% being strong producers, of which 88% carried the *gelE* and/or *efa* gene. Strong biofilm formation was correlated with higher MDR rates (51% in strong biofilm producers and 25% in weak producers), peaking at 58% in *E. faecalis* strong producers. Risk scoring classified up to 40% of isolates as high risk. These findings suggest that enterococci may contribute to food contamination and serve as potential reservoirs of resistance and virulence, underscoring the relevance of farm-level hygiene, rational antibiotic use, and targeted surveillance within a One Health framework.

**Supplementary Information:**

The online version contains supplementary material available at 10.1007/s11259-025-11061-8.

## Introduction

Enterococci are ubiquitous members of the gastrointestinal microbiota in humans and animals, and they are widely present in various environmental niches (Kim and Koo 2020). Due to their ability to tolerate harsh conditions such as high salt concentrations, low pH, and bile salts, some strains are intentionally used as starter or adjunct cultures in the fermentation of dairy, meat, and vegetable products. These technological properties, including bacteriocin production and proteolytic activity, contribute to flavor and microbial stability development in fermented foods (Kim and Koo 2020; Hansen et al. 2023; Codelia-Anjum et al. 2023). However, despite these beneficial roles, certain strains of *Enterococcus*, particularly *E. faecalis* and *E. faecium*, have emerged as significant opportunistic pathogens (WHO 2017; Codelia-Anjum et al. 2023). Their intrinsic resistance to multiple antibiotics and their ability to acquire additional resistance genes through horizontal gene transfer contribute to the global spread of multidrug-resistant strains. In clinical settings, vancomycin-resistant enterococci (VRE) pose a significant challenge due to limited therapeutic options and are listed among the most critical priority pathogens by the WHO (2017). The extent of antimicrobial resistance in enterococcal isolates is commonly assessed using the multiple antibiotic resistance (MAR) index, with values greater than 0.2 indicating exposure to high-risk sources of contamination. Notably, *E. faecium* is classified among the ESKAPE (*E. faecium*, *Staphylococcus aureus*,* Klebsiella pneumoniae*,* Acinetobacter baumannii*,* Pseudomonas aeruginosa*, and *Enterobacter* spp.) pathogens, which the WHO lists as high-priority organisms due to their multidrug resistance and association with healthcare settings (WHO 2017; Codelia-Anjum et al. 2023; Venkateswaran et al. 2023; Idris and Nadzir 2023). They are a common cause of endocarditis, urinary tract infections, gastrointestinal tract infections, and bacteremia in susceptible individuals (Noroozi et al. 2022; Rogers and Rice 2024). Most infections are intricately linked to biofilm formation, which enhances bacterial survival under hostile conditions, promotes adherence, and facilitates horizontal gene transfer (Byappanahalli et al. 2012; WHO 2022; Conwell et al. 2022). In addition to their antimicrobial resistance, the ability of enterococci to form biofilms and harbor virulence genes significantly contributes to their persistence in food production environments and increases the risk of foodborne infections (Conwell et al. 2022). These bacteria can carry various virulence factors, including the enterococcal surface protein (*esp*), gelatinase (*gelE*), endocarditis antigen A (*efaA*), and collagen-binding protein (*ace*), which contribute to bacterial adhesion, colonization, immune evasion, and biofilm formation. Specifically, the *esp* gene encodes a surface protein that promotes adherence and biofilm development; *gelE* encodes a gelatinase that hydrolyzes gelatin, collagen, and hemoglobin; ace encodes a protein that binds collagen and aids tissue adhesion; and *efaA* is linked to endocarditis pathogenesis (Franz et al. 2003; Anderson et al. 2016; Pillay et al. 2018; Ramos et al. 2020; Șchiopu et al. 2023; Ghazvinian et al. 2024).

It has been reported that livestock and contaminated food are sources of *Enterococcus*, particularly vancomycin-resistant *Enterococcus* (VRE) infection in humans (Kalpana et al. 2023). These bacteria can also transmit their antimicrobial resistance genes to humans directly through the food chain (Franz et al. 2003; Ghazvinian et al. 2024). Recent studies have reported high rates of VRE and multidrug resistance among enterococci isolated from food, animals, and clinical samples worldwide (Hansen et al. 2023; Kalpana et al. 2023; Ghazvinian et al. 2024). Given the increasing global concern regarding foodborne enterococci as reservoirs of antimicrobial resistance and virulence, their persistence through biofilm formation, and the limited number of farm-to-fork surveillance studies in Türkiye, this study was designed to provide an integrated assessment of *E. faecalis* and *E. faecium* along the meat production chain. Specifically, the aim was to determine their prevalence, antimicrobial resistance profiles, biofilm-forming capacity, and virulence gene distribution at different production stages, thereby identifying critical points where contamination and the spread of resistant strains may occur. The findings obtained from this work are intended to serve as baseline data for understanding the distribution and characteristics of enterococci along the meat production chain and to inform future One Health-oriented monitoring efforts.

## Materials and methods

### Sampling

This study was conducted in two meat processing plants, covering the stages from farm to meat products between September 2020 and June 2021. A total of 348 samples were collected across the farm-to-fork continuum, including 130 farm samples, 111 slaughterhouse samples, and 107 processing-plant samples. At each stage, samples were collected from animals, personnel, equipment, food-contact surfaces, carcasses, and final meat products. All samples were transported under cold-chain conditions and processed within 4 h. Detailed sampling categories and sample distribution are provided in Table 1.

**Table 1:** Distribution of *Enterococcus* spp., *E. faecalis*, and *E. faecium* isolated from different sample sources along the meat production chain

Sample sources	Number of Samples	Enterococcus spp. positive samples *n* (%)	E. faecalis *n* (%)	E. faecium *n* (%)
Farm	130	93 (72)	20 (22)	23 (25)
Cattle nasal swab	36	29 (81)	-	-
Cattle fecal swab	36	36 (100)	12 (33)	14 (39)
Personnel hand swap	8	-		
Personnel nasal swap	8	-		
Transport carts	11	11 (100)	3 (27)	4 (36)
Air	3	-	-	-
Feeders	14	8 (57)	2 (25)	3 (38)
Feed mixing machines	3	3 (100)	1 (33)	-
Surface	8	6 (75)	2 (33)	2 (33)
Spades	3	-	-	-
**Slaughterhouse**	**111**	**70 (63)**	**14 (20)**	**16 (23)**
Butcher hand swap	14	9 (64)	3 (33)	2 (22)
Butcher nasal swap	14	9 (64)	-	-
Stainless steel knives	14	7 (50)	3 (43)	2 (29)
Splitting saw	3	-	-	-
Surfaces	30	24 (80)	5 (21)	7 (29)
Carcass	36	21(58)	3 (14.3)	5 (14)
**Meat Plant**	**107**	**46 (43)**	**7 (15)**	**9 (20)**
Personnel hand swap	11	8 (73)	-	-
Personnel nasal swap	11	-	-	-
Mincing Machine	3	1 (33)	-	-
Filling Machine	3	1 (33)	-	-
Cutter	4	2 (50)	-	1 (50)
Surfaces	11	7 (64)	2 (29)	2 (29)
Sausage	24	8 (33)	3 (38)	-
Pastirma	22	12 (55)	-	5 (42)
Salami	18	7 (39)	2 (29)	1 (14)
**Total**	**348**	**209 (60)**	**41 (20)**	**48 (23)**

Percentages for *E. faecalis* and *E. faecium* indicate their proportions among *Enterococcus* spp.-positive isolates

The protocol secured approval from the Ethics Committees at Erciyes University, both the Human Research Ethics Committee (Approval No: 2019/155) and the Animal Experiments (Approval No: 19/036). All participating workers provided written informed consent and voluntarily agreed to participate. Participants confirmed that they had not received antimicrobial treatment within the preceding three months, and farm owners verified that animals had not been treated with antimicrobials during the previous month.

Nasal sampling in cattle was performed by inserting the swab 2–5 cm into one nostril and rotating it five times against the mucosa for 5–6 s (Gundog et al. 2025). In workers, hand swabs were taken from the forefingers and thumbs during working hours without prior notice, and nasal swabs were self-collected after instruction by inserting the swab into the nostril entrance and rotating it three times (Yildirim et al. 2020). All nasal and hand swabs from cattle and workers at farms, slaughterhouses, and meat-processing facilities were collected using sterile transportation swabs with Stuart medium (BTR, Ankara, Türkiye). Environmental swabs from farms, slaughterhouse, and processing-plant surfaces were collected in accordance with ISO 18,593, a standard that outlines validated procedures for surface sampling using contact plates or swabs (ISO 2004). Carcass sampling followed ISO 17,604, which specifies the recommended anatomical sites and surface areas for carcass swabbing. Accordingly, four 100 cm² regions (neck, breast, flank, and rump) on each half-carcass were sampled after the final wash and before chilling (ISO 2015). For final meat products (sausage, salami, and pastirma), approximately 50 g was aseptically collected per individual sample.

Air samples were collected using the passive settle plate method, following the principles described by Pasquarella et al. (2000), with minor modifications. In brief, Petri dishes containing Enterococcosel agar (EA; Becton Dickinson, USA) were exposed to ambient air for 20 min at approximately one meter above the ground surface in each sampling area. After exposure, the plates were covered and incubated at 37 °C for 24 h under aerobic conditions.

### Isolation of *Enterococcus* spp

Swab samples were placed in 5 mL of Enterococcosel broth (EB; Becton Dickinson, USA), and 10 g of each meat sample (sausage, pastirma, salami) was homogenized in 90 mL of EB. All samples were incubated at 35 °C for 24 h, after which 100 µL of pre-enrichment culture was streaked onto Enterococcosel agar (EA; Becton Dickinson, USA) and incubated at 35 °C for 24–48 h. Enterococci were isolated according to the ‘one colony per sample’ principle, with presumptive colonies characterized by a beige color and black halos on EA (Gundog et al. 2025). Presumptive *Enterococcus* isolates from selective agar were subcultured onto blood agar and confirmed by molecular analysis. Following genus-level identification, further analyses were restricted to *E. faecalis* and *E. faecium*, as these two species represent the primary Enterococcus lineages implicated in human infections.

### Identification of isolates by real-time PCR

Genomic DNA was extracted from presumptive Enterococcus isolates using the InstaGene Matrix DNA extraction kit (Bio-Rad, USA) according to the manufacturer’s instructions. The concentration and purity of the extracted DNA were assessed using a NanoDrop spectrophotometer (Thermo Scientific, USA). Genus-level identification was performed by real-time PCR targeting the 23 S rRNA gene due to its rapid and reliable performance in confirming Enterococcus isolates, as described in EPA Method 1611 (EPA, 2015) and Sanderson et al. (2019).

The qPCR reaction was performed in a final volume of 25 µL and included approximately 30–50 ng of genomic DNA (gDNA; 5 µL), 10 µL of Green SuperMix Low ROX (QuantaBio, USA), 0.5 µL of each primer (10 pmol), and 9 µL of RNase-free water. Amplification and melting curve analysis were performed using a CFX Connect Real-Time PCR Detection System (Bio-Rad, USA). Primer sequences and qPCR cycling conditions used in this study are provided in Table S1. A Ct cut-off value of 35 was employed as a conservative threshold based on EPA Method 1611 (EPA, 2015). Samples were considered positive for Enterococcus when the assay yielded a Ct value ≤ 35 with a single specific melt-curve peak, whereas no-template controls showed no amplification. *E. faecalis* ATCC 29,212 and *E. faecium* ATCC 8459 were included as positive controls for all PCR assays performed in this study.

### Identification of *E. faecalis* and ***E. faecium***, and profiling of vancomycin resistance and virulence genes

Species-level identification of *E. faecalis* and *E. faecium* was performed using a multiplex PCR assay targeting species-specific regions within the *ddl* (D-Ala–D-Ala ligase) genes. The *ddl* locus provides a reliable discriminatory marker for differentiating the two species. In the same multiplex reaction, glycopeptide resistance ligase genes (*vanA*, *vanB*, *vanC*) were amplified to detect acquired or intrinsic vancomycin resistance determinants, and additional PCR assays were used to screen for virulence-associated genes (*gelE*,* efaA*,* ace*,* asa1*,* espfs*,* espfm*).

Each 25 µL reaction mixture contained 12.5 µL of 2× DreamTaq Green PCR Master Mix (Thermo Fisher Scientific, USA), 1 µL of each primer (25 pmol), 2 µL of template DNA, and 8.5 µL of nuclease-free water. The oligonucleotide sequences and PCR amplification conditions used for all assays are detailed in Table S1.

Amplification products were separated on a 1.5% agarose gel prepared in 1× TBE buffer, electrophoresed at 120 V for 40 min, and visualized under UV illumination using a Gel Doc XR + system (Bio-Rad, USA).

### Antimicrobial susceptibility testing of isolates

Antimicrobial susceptibility of *E. faecalis* and *E. faecium* isolates was assessed by the Kirby–Bauer disc diffusion method using commercial antibiotic discs (Oxoid, Basingstoke, UK): ampicillin (10 µg), linezolid (30 µg), teicoplanin (30 µg), levofloxacin (5 µg), ciprofloxacin (5 µg), chloramphenicol (30 µg), tetracycline (30 µg), and erythromycin (15 µg). A bacterial suspension adjusted to the 0.5 McFarland standard was evenly inoculated onto the surface of Mueller–Hinton agar, and antibiotic discs were subsequently applied. The plates were then incubated at 37 °C for 18–24 h under aerobic conditions. After incubation, inhibition zone diameters were measured and interpreted according to CLSI breakpoints (CLSI 2024). The multiple antibiotic resistance (MAR) index for each isolate was calculated as the ratio between the number of antibiotics to which the isolate exhibited resistance and the total number of antibiotics tested in the study (Mir et al. 2022).

### Detection of Vancomycin resistance in isolates

Vancomycin susceptibility of isolates was assessed by broth microdilution according to ISO 20776-1:2019 (ISO 2019), which serves as the reference method recommended by EUCAST (2023). Briefly, two-fold serial dilutions of vancomycin (1–32 µg/mL) were prepared in Mueller–Hinton broth, and a 0.5 McFarland suspension of each isolate was diluted to achieve a final inoculum of 5 × 10⁵ CFU/mL (5 × 10⁴ CFU/well in 100 µL) in 96-well microtiter plates. The plates were incubated aerobically in a non-shaking incubator at 37 °C for 24 h. Quality control strains *E. faecalis* ATCC 29,212 (susceptible) and ATCC 51,299 (resistant) were included to ensure assay performance. After incubation, wells were visually assessed for turbidity by two independent and blinded observers, using the negative-control well as the reference for clarity; any turbidity comparable to the growth-control well was interpreted as bacterial growth, and the MIC was defined as the lowest vancomycin concentration showing no visible turbidity. Results were interpreted according to EUCAST (2023) breakpoints, with isolates classified as susceptible (≤ 4 µg/mL) or resistant (> 4 µg/mL).

### Quantitative assessment of biofilm formation

The biofilm-forming capacity of *E. faecalis* (*n* = 41) and *E. faecium* (*n* = 48) isolates was evaluated using 96-well flat-bottom sterile polystyrene microtiter plates. Isolates were first cultured in Tryptic Soy Broth supplemented with 2% glucose (gTSB; Merck, Germany) and incubated at 37 °C for 24 h. Cultures were then diluted 1:100 in fresh gTSB, and 100 µL of each dilution was inoculated into the wells of a sterile microtiter plate (Sigma Aldrich, Darmstadt, Germany) and incubated at 37 °C for a further 24 h. Negative controls consisted of wells containing only gTSB, whereas *Staphylococcus epidermidis* ATCC 35,984 served as the positive control. After incubation, wells were washed three times with 300 µL phosphate-buffered saline (PBS; pH 7.2), stained with 100 µL of 1% crystal violet for 30 min, rinsed three times with sterile distilled water, and air-dried at room temperature. Adherent cells were fixed with 150 µL of 99% ethanol for 15 min, after which the optical density (OD) was measured at 570 nm. Biofilm-forming ability was classified according to the criteria described by Stepanović et al. (2000). Isolates were categorized as non-producer (OD ≤ ODc), weak producer (ODc < OD ≤ 2×ODc), moderate producer (2×ODc < OD ≤ 4×ODc), and strong producer (OD > 4×ODc), where ODc was defined as the mean OD of the negative control plus three standard deviations. All assays were conducted in triplicate.

### Semi-quantitative risk profiling of *E. faecalis* and *E. faecium* isolates

Risk profiles of *E. faecalis* and *E. faecium* isolates were determined using a semi-quantitative scoring system (Krumperman 1983; EFSA 2015; Busschaert et al. 2010; Hernandez-Jover et al. 2021) incorporating four criteria: number of resistant antibiotics, MAR index, biofilm formation capacity, and presence of virulence genes. Biofilm formation capacity and virulence gene presence were scored as binary variables (0–1). The total risk score (RS) was calculated as:

$$\begin{array}{c}RS=2\left(number\;of\;resis\tan t\;antibiotics\right)+2\left(MAR\;Index\right)+\\1.5\left(Biofilm\right)+1.5\left(Virulence\;Genes\right)\end{array}$$

These criteria and weighting factors were selected to reflect their documented relevance to public health risk assessment, with antimicrobial resistance (resistance count and MAR index) emphasized as a major driver of pathogenic potential and dissemination risk (Krumperman 1983; EFSA 2015). Biofilm formation and virulence gene carriage were included due to their recognized roles in persistence, environmental survival, and infection severity (Busschaert et al. 2010; Hernandez-Jover et al. 2021).

Scores were classified as low (RS < 5), moderate (5 ≤ RS < 8), or high (RS ≥ 8), with thresholds corresponding to the 33rd and 67th percentiles of the dataset. Hierarchical clustering (Euclidean distance, single linkage) was applied to mean RS values across source categories (farm, slaughterhouse, processing plant) using Python 3.10 (SciPy).

### Statistical analysis

The associations between the isolation sources (farm, slaughterhouse and meat plant) and the prevalence of *Enterococcus* spp., *E. faecalis*, and *E. faecium* were analyzed using contingency tables. Pearson’s chi-square test was used for all comparisons; however, when more than 20% of the expected cell counts were less than 5, Fisher’s exact test was applied instead. Differences in biofilm formation between species, as well as associations between biofilm formation and antimicrobial resistance phenotypes, were assessed using the same categorical comparison tests based on data frequency. Risk Score (RS) values were expressed as mean ± standard deviation (SD) together with 95% confidence intervals (CI). Statistical significance was set at *p* < 0.05, and all analyses were conducted using The Jamovi Project v2.3.

## Results

**Fig. 1:**
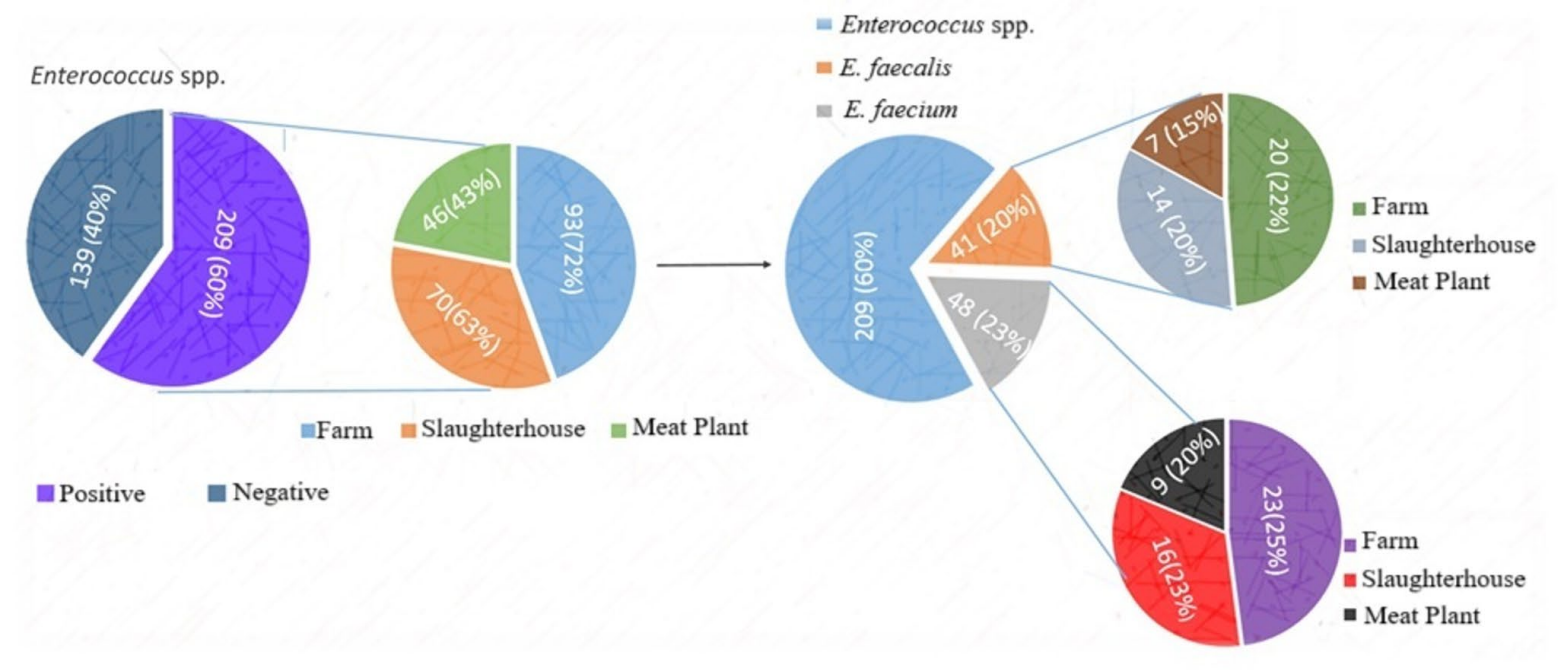
Distribution of *Enterococcus* spp., *E. faecalis*, and *E. faecium* Across Sampling Locations. The overall prevalence of *Enterococcus* spp. is illustrated, with 60% of the total samples testing positive. The distribution of positive samples across different sampling sources—farm, slaughterhouse, and meat plant—are shown. The distribution of *E. faecalis* and *E. faecium* among the positive samples highlights their respective proportions and the sources from which they were isolated. The farm was the most frequent source of both species, followed by the slaughterhouse and meat plan

*Enterococcus* spp. were detected in 60% (209/348) of all samples, with significantly higher prevalence in farm samples (72%, 93/130) than in slaughterhouse (63%, 70/111) and meat plant samples (43%, 46/107) (*p* < 0.001). Among these 209 *Enterococcus* isolates, 41 (20%) were identified as *E. faecalis* and 48 (23%) as *E. faecium*, while the remaining isolates were classified only at the genus level (Fig. 1). No significant differences in the distribution of *E. faecalis* and *E. faecium* were observed across the three environments (Pearson Chi-Square = 0.042, df = 2, *p* > 0.05). The distribution of *Enterococcus* spp. across production environments, sample types, and species is shown in Table 1. *E. faecium* was more frequently isolated from farm samples, particularly from cattle fecal swabs and feed-related equipment, whereas *E. faecalis* was more frequently recovered from meat plant isolates, including processed meat products and food-contact surfaces. Slaughterhouse isolates, obtained primarily from carcasses and processing equipment, exhibited a balanced distribution of both species.

All *E. faecalis* and *E. faecium* isolates were resistant to at least one tested antibiotic. In detail, out of 41 *E. faecalis* isolates, 27 (66%), 23 (56%), and 13 (32%) showed resistance to tetracycline, erythromycin, and chloramphenicol, respectively. In *E. faecium*, resistance to these antibiotics was observed in 33 of 48 isolates (69%), 28 (58%), and 19 (40%), respectively. Ciprofloxacin resistance was observed in 5 (12%) *E. faecalis* and 6 (13%) *E. faecium* isolates, and vancomycin resistance in 4 (10%) and 5 (10%) isolates, respectively; notably, all ciprofloxacin- and vancomycin-resistant isolates displayed MDR profiles (Table 2). In addition, all vancomycin-resistant isolates exhibited MIC values ≥ 16 µg/mL and carried the *vanA* gene. The *vanA* gene was also detected in 32 vancomycin-susceptible isolates, comprising 14 (34%) *E. faecalis* and 18 (38%) *E. faecium.* None of the isolates harbored *vanB* or *vanC* gene.

**Table 2:** Antimicrobial resistance profiles of *E. faecalis* and *E. faecium* isolates by biofilm formation category

Isolates	Antibiotic Resistance Profile n (%)					No of MDR isolates n (%)	Frequencies of MDR isolates n(%)	MDR Profile of Isolates	MAR Index
	C	CIP	E	TE	VAN				
***E. faecalis*** (*n* = 41)	13 (32)	5(12)	23 (56)	27 (66)	4 (10)	15 (37)	10 (67)	C + TE + E	0.33
							1 (7)	VAN + CIP + E	0.33
							3 (20)	VAN + CIP + TE	0.33
							1 (7)	CIP + C + TE + E	0.44
***E. faecium*** (*n* = 48)	19 (40)	6 (13)	28 (58)	33 (69)	5 (10)	20 (42)	14 (50)	C + TE + E	0.33
							2 (10)	VAN + CIP + C + E	0.44
							1 (5)	VAN + CIP + TE	0.33
							2 (10)	VAN + CIP + E	0.33
							1 (5)	CIP + C + TE + E	0.44

n: Number of isolates C: Chloramphenicol; CIP: Ciprofloxacin; E: Erythromycin; TE: Tetracycline; VAN: Vancomycin; MDR: Multi Drug Resistant

In total, 35 isolates (39%) were MDR (MAR index ≥ 0.33), comprising 15/41 (37%) *E. faecalis* and 20/48 (42%) *E. faecium* isolates. The predominant MDR profile was C + TE + E (24/35, 69%), including other combinations such as VAN + CIP + TE (4/35, 11%), VAN + CIP + E (3/35, 9%), and VAN + CIP + C + E (2/35, 6%) (Table 2).

All 89 isolates (41 *E. faecalis* and 48 *E. faecium*) produced biofilms, with 49 (55%) classified as strong and 40 (45%) as weak producers. When assessed by species, strong biofilm production was observed in 30 (63%) *E. faecium* isolates and 19 (46%) *E. faecalis* isolates, and this difference was not statistically significant (*p* > 0.05). Of the 89 biofilm-producing isolates, 78 (88%) carried at least one biofilm-associated gene. Among these, 46 isolates (52%) harbored both *gelE* and *efaA*, while 32 isolates (36%) carried only one of the two genes. In *E. faecium*, *gelE*,* efa*, and *gelE* + *efa* genes were detected in 9 (18%), 10 (21%), and 27 (56%) isolates, whereas in *E. faecalis* the rates were 5 (12%), 8 (20%), and 19 (46%), respectively (Table 3).

**Table 3:** Frequency, phenotypic and genotypic, of *E. faecalis* and *E. faecium* from the farm‑to‑fork continuum

Isolates	Source	No of isolates n (%)	Biofilm ability n (%)		Virulence genes n (%)			High-Risk Isolates n (%)	Mean RS (95% CI)	SD
			Strong	Weak	*efa*	*gelE*	*Efa + gelE*			
***E. faecalis***	**Farm (n = 93)**	20 (22)	9 (45)	11 (55)	3 (15)	2 (10)	10 (50)	7 (35)	5.87 (4.47–7.27)	2.99
	**Slaughterhouse (n = 70)**	14 (20)	7 (50)	7(50)	2 (14)	2 (14)	7 (50)	4 (29)	5.21 (3.78–6.64)	2.47
	**Meat Plant (n = 46)**	7 (15)	3 (43)	4(57)	3 (43)	1 (14)	2 (28)	2 (29)	5.52 (2.41–8.64)	3.37
	**Total (n = 209)**	**41 (20)**	**19 (46)**	**22 (60)**	**8 (20)**	**5 (12)**	**19 (46)**	**13 (32)**		
***E. faecium***	**Farm (n = 93)**	23 (25)	14 (56)	9(44)	4 (17)	5 (22)	13 (57)	9 (39)	6.18 (4.97–7.39)	2.79
	**Slaughterhouse (n = 70)**	16 (23)	12 (75)	4 (25)	2 (13)	4 (25)	9 (39)	6 (38)	6.46 (4.93–7.99)	2.87
	**Meat Plant (n = 46)**	9 (20)	4 (44)	5 (56)	4 (44)	-	5 (56)	4 (44)	6.36 (4.68–8.04)	2.19
	**Total (n= 209)**	**48 (23)**	**30 (63)**	**18 (37)**	**10 (21)**	**9 (18)**	**27 (56)**	**19(40)**		

Percentages were calculated based on the total number of isolates of each species (*E. faecalis* and *E. faecium*) within the corresponding source category. Mean RS, 95% CI and SD: Represents the average semi-quantitative risk score, 95% confidence interval and its standard deviation for isolates within each category

**Fig. 2:**
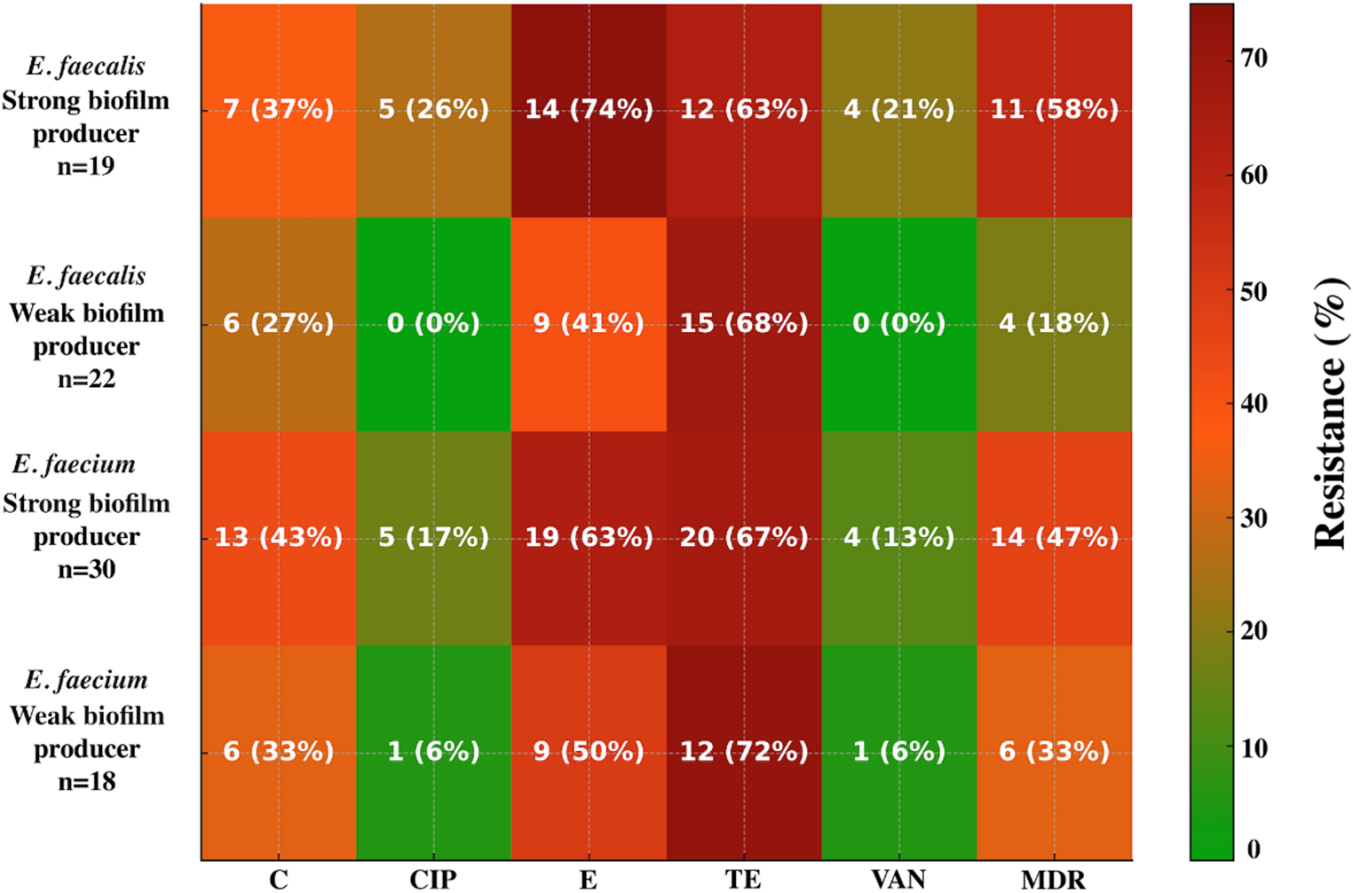
Heat map showing the antimicrobial resistance profiles of *E. faecalis* and *E. faecium* isolates categorized by biofilm-forming ability. Each cell displays the number and percentage of resistant isolates (n, %). The color scale (0–75%) ranges from green (low resistance) to dark red (high resistance). VAN: Vancomycin; CIP: Ciprofloxacin; TE: Tetracycline; C: Chloramphenicol; E: Erythromycin; MDR: Multi Drug Resistant

Notably, antimicrobial resistance and biofilm capacity were significantly associated, with MDR detected in 25 (51%) of 49 strong biofilm producers compared with 10 (25%) of 40 weak producers (Pearson Chi-Square = 6.25, df = 1, *p* < 0.05). MDR was more frequently observed among strong biofilm producers in both species. In *E. faecalis*, 58% (11/19) of strong biofilm producers were MDR, compared with 18% (4/22) of weak producers, while in *E. faecium* the corresponding rates were 47% (14/30) and 33% (6/18), respectively. Strong producers also demonstrated higher resistance to ciprofloxacin, erythromycin, and vancomycin, whereas tetracycline and chloramphenicol resistance remained similar between strong and weak biofilm-producing isolates (Fig. 2).

According to the semi-quantitative risk scoring system, high-risk classification was assigned to 13/41 (32%) of *E. faecalis* and 19/48 (40%) of *E. faecium* isolates. Mean risk scores by source indicated a moderate-risk category for all groups (Table 3).

**Fig. 3:**
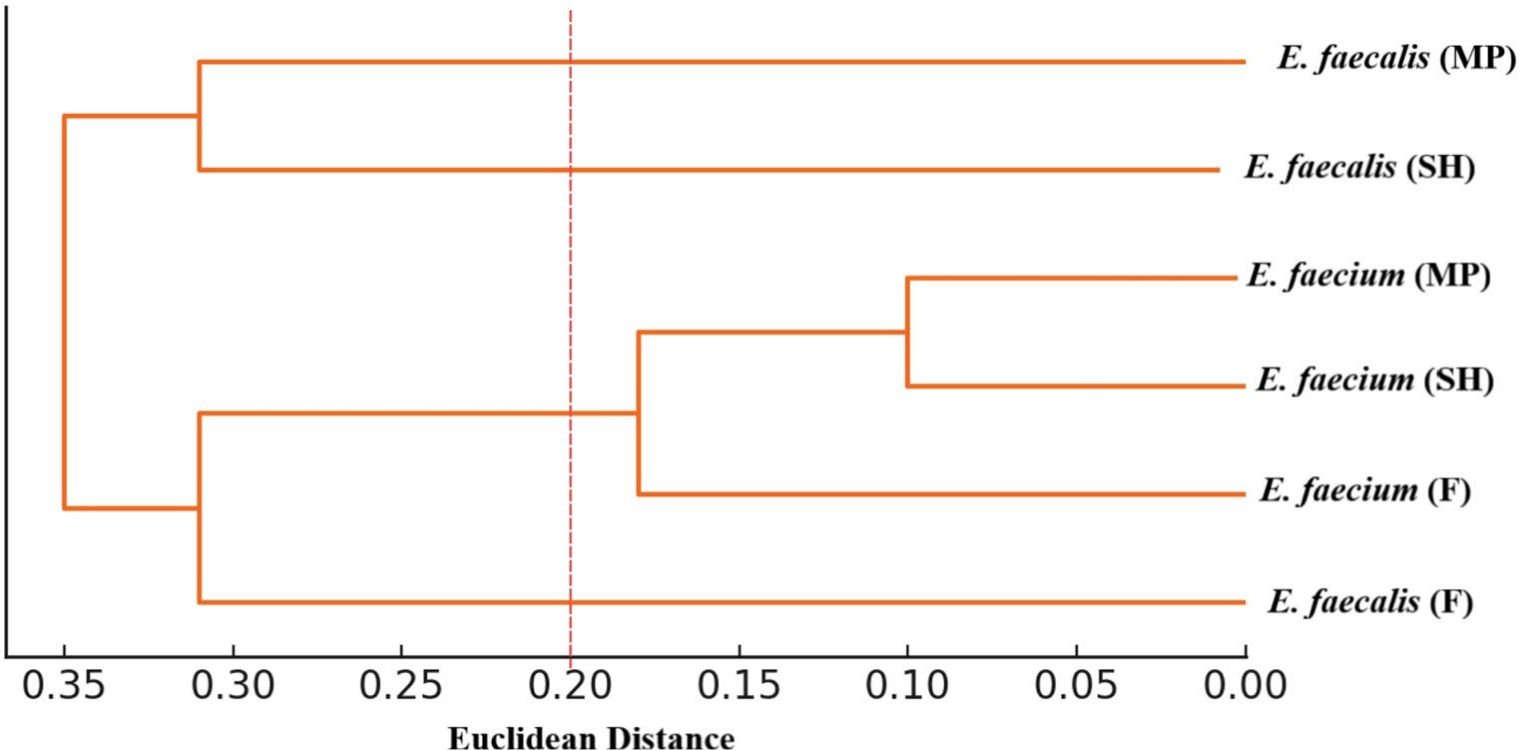
Hierarchical clustering of *E. faecalis* and *E. faecium* isolate groups (farm, slaughterhouse, meat-processing facility) based on mean semi-quantitative risk scores. Clustering was performed using Euclidean distance and the single-linkage method. A cut-off value of 0.20 (dashed line) was applied to define the main clusters. Horizontal branch lengths represent intergroup distance (longer branches = greater dissimilarity; shorter branches = greater similarity). MP: meat plant; SH: slaughterhouse; F: farm

Hierarchical clustering based on mean semi-quantitative risk scores revealed three main clusters: *E. faecium* isolates from farm, slaughterhouse, and meat plants formed a tight cluster (scores: 6.18–6.46), while *E. faecalis* isolates from slaughterhouse (5.21) and meat plants (5.52) clustered together before merging with the farm *E. faecalis* group (5.87), which subsequently joined the *E. faecium* cluster (Fig. 3).

## Discussion

Enterococci, integral members of the autochthonous gastrointestinal microbiota in mammals, can shift from commensals to opportunistic pathogens due to their ability to translocate across intact intestinal epithelium (Golob et al. 2019; Rogers and Rice 2024). *E. faecalis* and *E. faecium* are currently the third leading cause of nosocomial infections, notable for their multidrug resistance (MDR) and virulence determinants. Foods of animal origin may act as reservoirs and transmission vehicles for MDR enterococci and antimicrobial resistance (AMR) genes (Hernando-Amado et al. 2019; Boccella et al. 2021; Cattoir 2022; Rogers and Rice 2024; Cebeci 2024). Dissemination of MDR strains within the production environment can occur through direct animal contact, environmental persistence, and handling or consumption of contaminated products (Cattoir 2022; Grudlewska-Buda et al. 2023). Therefore, surveillance of MDR enterococci along the farm-to-fork continuum is crucial for implementing targeted control measures and guiding regulatory frameworks.

In the present study, *E. faecalis* and *E. faecium* accounted for 21% and 23%, respectively, of the samples yielding *Enterococcus* spp. (60%), consistent with previous reports documenting their isolation at different prevalence levels from foods of animal origin (Kojima et al. 2010; Jackson et al. 2011; Aslam et al. 2012; Różańska et al. 2015; Jans et al. 2018; de Jong et al. 2019; Kim et al. 2021; Morris et al. 2023). Across the production chain, the highest recovery rate of *Enterococcus* spp. (72%), comprising *E. faecalis* (22%) and *E. faecium* (25%), was observed at the farm stage. At this stage, *E. faecium* predominated in high-risk sources such as cattle feces (39%) and feed-related equipment (33–36%), while *E. faecalis* was more frequent on environmental surfaces (38%). These findings may support the role of farms as important contamination sources.

Environmental dissemination through animal waste and subsequent contamination during slaughter and processing has also been reported in previous studies (Manyi-Loh et al. 2018; Badul et al. 2021). In this study, slaughterhouses represented the second major contamination stage (63%), with similar proportions of *E. faecalis* (20%) and *E. faecium* (23%), most frequently detected on carcasses (33–54%) and processing tools such as butcher hand swabs (22%) and stainless-steel knives (29%). These findings suggest that carcass contamination may be linked to handling practices and hygiene conditions, consistent with previous reports of slaughter-stage contamination (Wambui et al. 2018; Telli et al. 2021; Mansour et al. 2023; Cebeci 2024). During meat processing, the prevalence declined to 43%, with *E. faecalis* being more common in processed meat products, such as pastirma (42%) and salami (29%), whereas *E. faecium* was more frequent in sausages (27%). This distribution may reflect differences in species occurrence along the production continuum, with *E. faecium* more frequently detected in farm-associated samples and *E. faecalis* more commonly observed in post-slaughter environments and finished products. Furthermore, although contamination decreased along the chain, the detection of enterococci in final products and on contact surfaces may indicate limitations in hygiene measures and a continued risk of cross-contamination. Similar findings have been reported in beef processing plants and beef products (Aslam et al. 2010; Holman et al. 2021; Cebeci 2024).

Antimicrobial susceptibility testing revealed that both *E. faecalis* and *E. faecium* displayed the highest resistance to tetracycline (66% and 69%, respectively) and erythromycin (56% and 58%, respectively), extensively used in both veterinary and human medicine, particularly for enteric infections (Caneschi et al. 2023). This widespread resistance may be associated with frequent therapeutic and prophylactic use, including reported use in livestock production, as well as potential hygiene limitations during processing. Comparable resistance levels have been reported in human isolates from Türkiye, with tetracycline resistance of 72.9% and 36.4% and erythromycin resistance of 64.7% and 91.2% for *E. faecalis* and *E. faecium*, respectively (Kilbas and Ciftci 2018). AMR to tetracycline is frequently linked to co-selection of resistance to critically important antimicrobials (CIAs) for human medicine (Klibi et al. 2015; Fossen et al. 2023, 2024), which may raise public health concerns. Previous studies have documented erythromycin and tetracycline resistance rates in animal-derived enterococci ranging from 0 to 100% and 3–67.5%, respectively (Aslam et al. 2012; Tyson et al. 2018; Davedow et al. 2020; Makarov et al. 2022; Cebeci 2024).

Ciprofloxacin resistance was detected in 12–13% of isolates, all of which were MDR and exhibited resistance in combination with other antimicrobials, most frequently tetracycline and erythromycin. Ciprofloxacin is classified as a critically important antimicrobial by the World Health Organization (WHO 2017). Comparable resistance levels in *E. faecium* from livestock and animal products (1.1–55%) have been reported (Hershberger et al. 2005; Kim and Koo 2020; Holman et al. 2021; Kim et al. 2021; Fossen et al. 2024). While no resistance was detected to ampicillin, levofloxacin, or linezolid, vancomycin resistance was present in 10% of isolates from both species. Similar to ciprofloxacin, vancomycin resistance also occurred exclusively in MDR profiles, frequently in combination with ciprofloxacin, tetracycline, and/or erythromycin. Vancomycin-resistant enterococci have been isolated from food animals, retail meat, vegetables, and drinking water (Tansuphasiri et al. 2006; Molale and Bezuidenhout 2016; Foka and Ateba 2019). Vancomycin resistance is of particular concern for *E. faecium*, which the WHO classifies as a high-priority pathogen in urgent need of new therapeutic options (Makarov et al. 2022). In Türkiye, resistance rates in human isolates have ranged from 1.0 to 2.2% for *E. faecalis* and 10.3–11.3% for *E. faecium* (Kilbas and Ciftci 2018). Global evidence links vancomycin resistance in enterococci to avoparcin use as a growth promoter in animal husbandry (Bager et al. 1997; Foka and Ateba 2019; Bortolaia and Guardabassi 2023), suggesting that such resistance patterns may be influenced by historical avoparcin use or by co-selection driven by other antimicrobials (Molale and Bezuidenhout 2016; Foka and Ateba 2019).

It is noteworthy that all isolates in this study demonstrated biofilm-forming ability, with 55% classified as strong biofilm producers. The notably higher ciprofloxacin resistance observed in strong biofilm-producing *E. faecalis* isolates (26%), together with the absence of resistance among weak producers, has been previously associated in earlier studies with the extracellular matrix of mature biofilms, which can impede antimicrobial penetration and create localized microenvironments that favor the survival and maintenance of resistant phenotypes (Grudlewska-Buda et al. 2023; Ghazvinian et al. 2024). Given that ciprofloxacin is classified as a critically important antimicrobial by the WHO, the presence of resistant strains within robust biofilm structures may pose a potential risk for dissemination along the meat production chain. A similar pattern was observed for erythromycin and vancomycin in *E. faecalis*, where strong producers exhibited resistance rates of 74% and 21%, respectively. The association between strong biofilm formation and vancomycin resistance is noteworthy, as it mirrors observations suggesting that biofilm development may contribute to increased tolerance to glycopeptides by providing a protective microenvironment and facilitating the persistence of resistant phenotypes. Such co-occurrence might be facilitated by horizontal gene transfer events within biofilms, which facilitate the exchange of resistance determinants among sessile cells (Conwell et al. 2022). The higher resistance rates to ciprofloxacin (17%), erythromycin (63%), and vancomycin (13%) in strong biofilm-producing *E. faecium* isolates than in weak producers (6%, 50%, and 6%, respectively) further support an association between biofilm-forming capacity and antimicrobial resistance. Moreover, tetracycline resistance remained uniformly high in both *E. faecium* (67% in strong and 72% in weak biofilm producers) and *E. faecalis* (63% in strong and 68% in weak producers), likely reflecting long-term selective pressure from its extensive use in livestock production rather than any biofilm-associated effect. Chloramphenicol resistance also followed the strong–weak pattern, albeit at lower prevalence, suggesting that even for antimicrobials with reduced contemporary usage, biofilm-associated persistence mechanisms may still confer a survival advantage.

The higher prevalence of MDR among strong biofilm producers (*E. faecalis*: 58% in strong producers and 18% in weak producers; *E. faecium*: 47% in strong producers and 33% in weak producers) suggests that these isolates may pose a potential public health concern. These strains combine resistance to multiple antimicrobial classes with the ability to persist on contact surfaces in slaughterhouses and processing plants, which may increase the likelihood of cross-contamination between carcasses and meat products. In addition, the high prevalence of *gelE* and *efa* genes, particularly when present in combination, is consistent with a genetic contribution to biofilm formation (Moraes et al. 2012; Klibi et al. 2015; Foka and Ateba 2019; Gungor et al. 2023). However, their detection in weak biofilm producers indicates that gene presence alone does not determine biofilm strength; expression levels, environmental conditions, and strain physiology are also likely to influence the phenotype.

Semi-quantitative risk scoring classified 32% of *E. faecalis* and 40% of *E. faecium* isolates as high risk, with the latter’s elevated pathogenic potential being consistent with its greater resistance burden and hospital-adapted traits. Although mean risk scores across sources remained in the moderate category, high-risk isolates were dispersed across all stages of the production chain, suggesting the absence of a single critical point. Hierarchical clustering suggested genetic homogeneity among *E. faecium* isolates across sources, whereas *E. faecalis* showed source-specific divergence, with farm-derived isolates eventually clustering with *E. faecium*. These findings underscore the need for comprehensive hygiene and control measures that encompass the entire supply chain, rather than focusing on isolated points. In this context, RS, despite being a semi-quantitative indicator, provided useful insight into which stages along the farm-to-fork chain the isolated strains may carry greater importance. The higher RS values observed for *E. faecium* are consistent with the resistance burden identified in this species and indicate that, despite its lower prevalence, it may warrant closer attention in risk evaluations. In contrast, the more variable RS distribution among *E. faecalis* isolates and their source-dependent clustering patterns suggest that contamination dynamics may differ across stages of the chain, potentially reflecting more localized influences at the farm level. Therefore, RS offers a useful complementary tool for interpreting the relative importance of isolates and for holistically assessing their potential risk contributions within the farm-to-fork framework. Similarly, recent applications of semi-quantitative tools in constructing hazard–product–process risk profiles for meat chains have demonstrated that these approaches can offer a practical and structured means of ranking hazards and prioritizing control strategies when only partial or heterogeneous data are available (Presi et al. 2009; Hernandez-Jover et al. 2021; Crotta et al. 2022). As also highlighted in recent international guidance, semi-quantitative approaches can provide a structured and practical basis for comparing microbiological and AMR-related risks across multiple sources, particularly when the data is heterogeneous or insufficient for fully quantitative modeling, thereby supporting their use as complementary tools in farm-to-fork risk profiling (FAO 2020; WHO 2021).

While the current study was successful in providing baseline data on the prevalence, antimicrobial resistance, biofilm formation, and virulence traits of *E. faecalis* and *E. faecium* along the meat production chain in Kayseri, Türkiye, there are limitations to the findings, primarily due to limited resources. Firstly, the sample size of the current study was limited, as sampling could only be conducted in two high-capacity facilities located in Kayseri that voluntarily granted permission. Although these plants are representative of large-scale production in the region, this limitation reduces the power of comparison and limits the generalizability of the findings. Secondly, the cross-sectional design did not allow assessment of temporal trends in resistance patterns within individual hosts, workers, or environments over time. Third, analyzing a single isolate per sample might not fully reflect the potential diversity of *E. faecalis* and *E. faecium* populations and their antimicrobial resistance profiles within a sample. Finally, the lack of molecular typing of isolates using advanced techniques such as PFGE, MLST, or WGS limited the ability to resolve clonal relationships and trace potential transmission pathways. Despite these limitations, the study provides useful baseline data that may inform future research and surveillance.

## Conclusion

This study indicated that *E. faecalis* and *E. faecium* persist throughout the meat production continuum, including in final products, which may have implications for food safety and public health. Most isolates were resistant to at least one antimicrobial agent, and 39% exhibited multidrug resistance. Although resistance to critically important agents such as ciprofloxacin and vancomycin occurred at low frequencies, its detection among strong biofilm-producing isolates may increase the risk of enhanced environmental persistence and increased transmission along the food production chain. The detection of resistant isolates across different stages of the production chain suggests that both livestock and processing environments could represent potential sources. At the same time, meat products may play a role in the dissemination of antimicrobial resistance, underscoring the relevance of a One Health perspective. These findings highlight the importance of integrated control strategies, including antimicrobial stewardship in primary production, strict hygiene during slaughter and processing, and effective sanitation practices to mitigate biofilm formation. Moreover, future research incorporating larger, multi-regional cohorts and genome-based and molecular typing approaches would be valuable for better understanding the resistance and persistence mechanisms of Enterococcus in meat production environments.

## Supplementary Information

Below is the link to the electronic supplementary material.

Supplementary Material 1 (DOCX 21.3 KB)

## Data Availability

No datasets were generated or analysed during the current study.
