# Supplementary material for: Tracking persistent and resistant Enterococcus faecalis and E. faecium from farm to fork: biofilm-linked risks in antibiotic resistance of isolates
Source: Vet Res Commun. 2026 Jan 15;50(2):100. doi: 10.1007/s11259-025-11061-8 (PMC12808194; doi:10.1007/s11259-025-11061-8)
Supplement: Supplementary file 1 — Supplementary Material 1 (DOCX 21.3 KB) [file 11259_2025_11061_MOESM1_ESM.docx]

**Table S1.** Primer sequences and PCR conditions for species identification, vancomycin resistance and virulence gene detection in E. faecalis and E. faecium.

| **Gene Type** | **Genes** | **Sequence (5’-3’)** | **PCR Conditions** | | | | | | **Size (bp)** | **References** |
| --- | --- | --- | --- | --- | --- | --- | --- | --- | --- | --- |
|  | | | **Initial Denaturation** | **Denaturation** | **Annealing** | **Extension** | **Final extension** | **Cycle** |  |  |
| **Control** | **23SRNA** | GAGAAATTCCAAACGAACTTG  CAGTGCTCTACCTCCATCATT | 95°C /10 min | 95°C /15 sec | 60°C/1min | 72°C /1min | - | 40 | 93 | Sanderson et al. 2019  Gungor et al., 2023  Dutka-Malen et al.1995 |
|  | ***ddl_E. faecalis_*** | CACCTGAAGAAACAGGC  ATGGCTACTTCAATTTCACG | 94°C /3 min | 94°C /1 min | 54°C/1min | 72°C /1min | 72°C /7min | 30 | 475 |  |
|  | ***ddl_E. faecium_*** | GCAAGGCTTCTTAGAGA  CATCGTGTAAGCTAACTTC | 94°C /3 min | 94°C /1 min | 48°C/1min | 72°C /1min | 72°C /7min | 30 | 550 |  |
| **Resistance genes** | ***vanA*** | GGGAAAACGACAATTGC  GTACAATGCGGCCGTTA | 94°C /3 min | 94°C /1 min | 52°C/1min | 72°C /1min | 72°C /7min | 30 | 732 | Dutka-Malen et al.1995 |
|  | ***vanB*** | ATGGGAAGCCGATAGTC  GATTTCGTTCCTCGACC | 94°C /3 min | 94°C /1 min | 53°C/1min | 72°C /1min | 72°C /7min | 30 | 635 |  |
|  | ***vanC*** | GGTATCAAGGAAACCTC  CTTCCGCCATCATAGCT | 94°C /3 min | 94°C /1 min | 50°C/1min | 72°C /1min | 72°C /7min | 30 | 822 |  |
| **Virulence Genes** | ***Ace*** | GGAATGACCGAGAACGATGGC  GCTTGATGTTGGCCTGCTTCCG | 94°C /3 min | 94°C /1 min | 62°C /1min | 72°C /1min | 72°C /7min | 30 | 616 | Creti et al. 2004 |
|  | ***esp_fs_*** | TTGCTAATGCTAGTCCACGACC  GCGTCAACACTTGCATTGCCGAA |  |  |  |  |  |  | 933 | Reviriego et al. 2005 |
|  | ***esp_fm_*** | TTGCTAATGCAAGTCACGTCC  GCATCAACACTTGCATTACCGAA | 94°C /3 min | 94°C /1 min | 59°C/1min | 72°C /1min | 72°C /7min | 30 | 955 | Reviriego et al. 2005 |
|  | ***asa1*** | GCACGCTATTACGAACTATGA  TAAGAAAGAACATCACCACGA |  |  |  |  |  |  | 375 | Vankerckhoven et al. 2004 |
|  | ***gelE*** | TATGACAATGCTTTTTGGGAT  AGATGCACCCGAAATAATATA | 94°C /3 min | 94°C /1 min | 52°C/1min | 72°C /1min | 72°C /7min | 30 | 213 | Vankerckhoven et al. 2004 |
|  | ***efaA_fs_*** | GACAGACCCTCACGAATA  AGTTCATCATGCTGTAGTA |  |  |  |  |  |  | 705 | Eaton and Gasson 2001 |
|  | ***efaA_fm_*** | AACAGATCCGCATGAATA  CATTTCATCATCTGATAGTA |  |  |  |  |  |  | 735 |  |
